# A Case of Gastrocolic Cutaneous Fistula in a Patient With Crohn’s Disease

**DOI:** 10.7759/cureus.115099

**Published:** 2026-08-24

**Authors:** McKenzie Clapp, Kellen Welch

**Affiliations:** 1 General Surgery, Summa Health, Akron, USA; 2 Colorectal Surgery, Summa Health, Akron, USA

**Keywords:** crohn's disease, crohn's fistula, gastrocolic fistula, internal fistula, surgical management of crohn's disease

## Abstract

Spontaneous gastrointestinal fistula formation is often due to Crohn’s disease (CD). Though most CD fistulas are perianal in nature, intra-abdominal internal fistulas can develop as well. Here we present a rare case of a gastrocolic cutaneous fistula in a patient with long standing CD. The fistula required a two-stage surgical approach. The first procedure addressed the acute cutaneous abscess portion of the fistula and the second procedure managed the gastrocolic portion. This case demonstrates the complex surgical management involved in taking care of CD patients with internal fistulas.

## Introduction

A fistula is an epithelized connection between two surfaces and can be classified into internal and external. An internal fistula is a connection between internal organs whereas an external fistula is a connection between an organ and the skin. The most common fistula etiologies include trauma, surgical sites, and Crohn’s disease (CD) [1].

CD is the most common cause of spontaneous gastrointestinal fistulas in developed nations. The risk of fistula formation following initial CD diagnosis is approximately 30% at 10 years and 50% at 20 years [2,3]. Of CD fistulas, previous studies have shown 54% to be perianal, 24% enteroenteric, 9% rectovaginal and 13% involving other anatomic locations [3,4]. Gastrocolic fistulas in CD are rare, with less than 100 cases reported [5]. Despite advancements in medical therapy, these fistulas often require surgery.

Here we present a rare case of a gastrocolic-cutaneous fistula in a patient with CD. We further discuss the surgical management involved in resection of this complex fistula.

This case report was previously presented as a poster at the 2025 Society of American Gastrointestinal and Endoscopic Surgeons Annual Meeting on March 14, 2025.

## Case presentation

**Figure 1:**
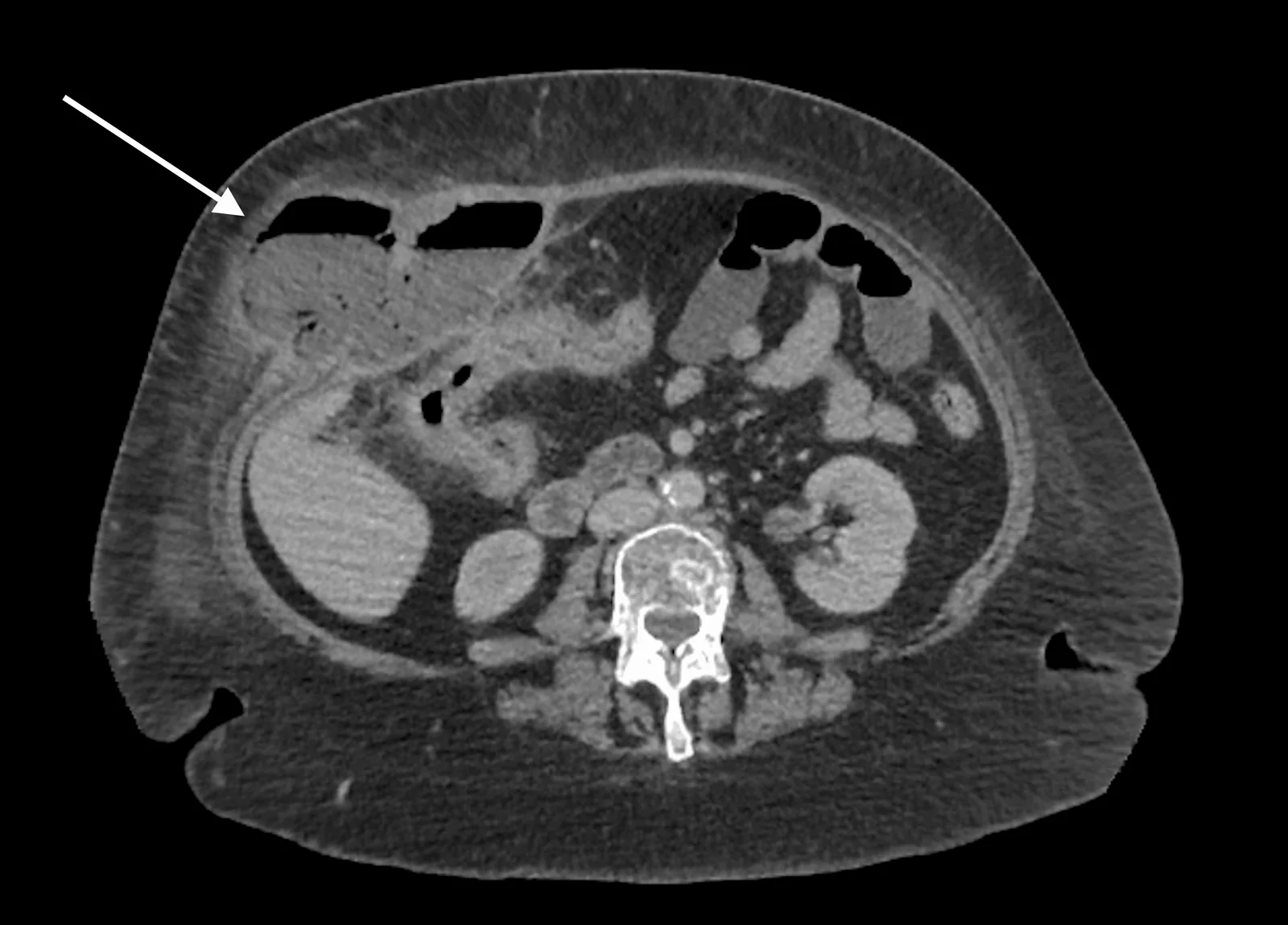
CT scan abdomen and pelvis with intravenous contrast, with arrow pointing to acute cutaneous abscess portion of fistula

A 54-year-old female patient with a past medical history of CD presented to the emergency department with three months of a painful, right-sided abdominal mass. The patient’s CD had been diagnosed via biopsy 30 years prior and was managed only with intermittent prednisone for acute flares. Imaging was obtained and showed a 13 x 7.2 cm subcutaneous abscess in the right anterolateral abdominal wall, focal inflammatory changes, and colitis involving the adjacent hepatic flexure, consistent with a fistulous process (Figure 1). The patient was then taken to the operating room for incision and drainage of the acute abscess and a large abscess cavity was explored without any obvious fistula opening visualized. The patient was discharged home and definitive operative resection of her fistula was deferred. 

The patient was seen by outpatient gastroenterology and given one dose of adalimumab in attempt to medically control her CD prior to definitive surgery. Months later, she was seen in the colorectal clinic and endorsed continuous daily feculent drainage from her colocutaneous fistula site that was difficult for her to manage at home. The patient had received only one dose of adalimumab months prior and further doses were held. She was scheduled for a robotic-assisted right colectomy with takedown of the colocutaneous fistula approximately five months after her initial presentation.

In the operating room, robotic lysis of adhesions and takedown of the colocutaneous portion of the fistula was successfully completed. Normal anatomy was restored and the ileocolic vessels were divided in standard fashion with a 60 mm robotic stapler. A proximal endpoint was made with the robotic stapler at the distal ileum. While evaluating the transverse colon for a distal transection point, it became extremely difficult to mobilize due to the weight and size of the specimen and significant adhesions to the duodenum. The case was converted to an open procedure and the colon and small bowel were extra-corporealized via a midline laparotomy incision. A clear gastrocolic fistula was visualized. A partial gastric resection of the greater curvature of the stomach was completed with a 75 mm linear stapler. After this, a safe distal transection point was created on the mid transverse colon and the colon and stomach were sent en-bloc as a specimen to pathology. An isoperistaltic ileocolonic anastomosis was created. The abdomen was then closed in standard fashion and the cutaneous fistula site was further debrided and irrigated. 

The patient’s post-operative course was prolonged due to pain control and ileus. After gradual tolerance of diet advancement, she was discharged home on post-operative day 10. At her one week follow-up appointment, she had significant improvement in her pain. 

Final pathology showed chronic active enteritis and colitis with transmural lymphoid aggregates, fibrosis, ulceration, serositis and a gastrocolic fistula consistent with CD.

## Discussion

The diagnosis and management of patients with CD gastrocolic fistulas can be complex. Initial symptoms can include diarrhea with undigested food matter or fecal emesis. However, only approximately 30% of patients will have these characteristic symptoms [6]. Due to the often vague presentation of these fistulas, pre-operative imaging is important for diagnosis and operative planning. Seastedt et al. demonstrated that CT enterography and MRI enterography accurately identify abscesses in CD patients with a 90% and 95% accuracy rate, respectively [7]. Fistula and stenosis were identified less accurately with both modalities, and unexpected intra-operative findings led to changes in the surgical procedure in 26% of cases [7,8]. This is consistent with our patient’s case, in which a cutaneous abscess was readily identified on pre-operative imaging, but evidence of a gastrocolic fistula was not clear and the surgical operation had to be altered due to intra-operative findings. 

Most CD gastrocolic fistulas will require operative intervention. Laparoscopic and robotic surgery have been demonstrated as safe surgical options in CD patients [9,10]. Conversion from laparoscopic to open surgery in CD patients typically ranges from 8.5% to 13.4% [10]. In operations for complex or recurrent disease the rate of conversion increases to 25-42% [10,11]. An internal fistula is an example of complex disease and is a risk factor for conversion due to the technical difficulty required to adequately address the fistula [12]. 

In this case, the thickened and bulky nature of the gastrocolic fistula required conversion to an open procedure to adequately mobilize and resect the fistula. Often gastrocolic fistulas can be addressed with a simple partial resection of the stomach in conjunction with resection of the colonic component. The gastric portion of our patient’s fistula was resected with a linear stapler, removing only a small portion of the greater curvature of the stomach. A similar approach was used in Nagai et al., in which a linear stapler was used to successfully address multiple CD gastrocolic fistulas laparoscopically [13]. This approach proved effective and efficient in both our case and for Nagai et al. 

This case demonstrates the complex nature of CD internal fistulas and how the surgical approach has to be individualized to the patient’s specific disease. 

## Conclusions

Gastrocolic fistulas are a rare type of internal fistula seen in CD and typically require operative intervention. In this case report of a gastrocolic cutaneous fistula in a patient with CD, we discuss our two-stage surgical approach. This included first addressing an acute cutaneous abscess, followed by a delayed partial gastric resection and extended right colectomy. This case demonstrates the complex surgical management involved in addressing internal fistulas in patients with CD.
